# Red blood cell transfusion in septic shock - clinical characteristics and outcome of unselected patients in a prospective, multicentre cohort

**DOI:** 10.1186/1757-7241-22-14

**Published:** 2014-02-27

**Authors:** Ragnhild G Rosland, Marte U Hagen, Nicolai Haase, Lars B Holst, Morten Plambech, Kristian R Madsen, Peter Søe-Jensen, Lone M Poulsen, Morten Bestle, Anders Perner

**Affiliations:** 1 Department of Intensive Care, Copenhagen University Hospital, Rigshospitalet, Denmark; 2 Intensive Care Unit, Copenhagen University Hospital, Hvidovre, Denmark; 3 Department of Intensive Care, Holbæk Hospital, DK-4300 Holbæk, Denmark; 4 Department of Intensive Care, Næstved Hospital, Næstved, Denmark; 5 Department of Intensive Care, Copenhagen University Hospital, Herlev, Denmark; 6 Department of Anaesthesia and Intensive Care, Køge Hospital, Køge, Denmark; 7 Department of Anaesthesia and Intensive Care Medicine, Hillerød Hospital, Hillerød, Denmark

**Keywords:** Intensive care, Sepsis, Shock, Blood transfusion, Mortality

## Abstract

**Background:**

Treating anaemia with red blood cell (RBC) transfusion is frequent, but controversial, in patients with septic shock. Therefore we assessed characteristics and outcome associated with RBC transfusion in this group of high risk patients.

**Methods:**

We did a prospective cohort study at 7 general intensive care units (ICUs) including all adult patients with septic shock in a 5-month period.

**Results:**

Ninety-five of the 213 included patients (45%) received median 3 (interquartile range 2–5) RBC units during shock. The median pre-transfusion haemoglobin level was 8.1 (7.4–8.9) g/dl and independent of shock day and bleeding. Patients with cardiovascular disease were transfused at higher haemoglobin levels. Transfused patients had higher Simplified Acute Physiology Score (SAPS) II (56 (45-69) vs. 48 (37-61), p = 0.0005), more bleeding episodes, lower haemoglobin levels days 1 to 5, higher Sepsis-related Organ Failure Assessment (SOFA) scores (days 1 and 5), more days in shock (5 (3-10) vs. 2 (2-4), p = 0.0001), more days in ICU (10 (4-19) vs. 4 (2-8), p = 0.0001) and higher 90-day mortality (66 vs. 43%, p = 0.001). The latter association was lost after adjustment for admission category and SAPS II and SOFA-score on day 1.

**Conclusions:**

The decision to transfuse patients with septic shock was likely affected by disease severity and bleeding, but haemoglobin level was the only measure that consistently differed between transfused and non-transfused patients.

## Background

Septic shock is characterized by inflammatory-induced circulatory failure leading to organ failure and high mortality rates [1]. Antibiotics, source control and resuscitation with fluids and vasopressor and inotropic agents are the mainstay of treatment for septic shock and may be supplemented with transfusion of red blood cells (RBCs) in the case of anaemia to sustain sufficient oxygen delivery [2].

The use of RBC transfusion is, however, controversial. The Surviving Sepsis Campaign (SSC) guidelines for the management of severe sepsis and septic shock distinguish between early (< 6 hours) and later (> 6 hours) stages of septic shock. During the first 6 hours of resuscitation of septic shock transfusion to a haematocrit above 30% (approx. 10 g/dl) is recommended if central venous oxygen saturation (ScvO_2_) remains below 70% despite initial fluid and vasopressor therapy. In the later stage, transfusion is recommended when haemoglobin is less than 7.0 g/dl to a level of 7.0 to 9.0 g/dl. In extenuating circumstances, such as severe hypoxaemia, ischaemic coronary artery disease or acute haemorrhage, patients may be transfused at a higher level of haemoglobin [2].

The guidelines are based on two randomized clinical trials (RCTs). The Early Goal-Directed Therapy (EGDT) trial indicated improved outcome with a relatively liberal transfusion strategy as part of a complex treatment protocol [3]. In contrast, the Transfusion Requirements in Critical Care (TRICC) trial showed no significant difference in 90-day mortality when comparing a liberal and a restrictive transfusion strategy. However, trial results indicated lower mortality with the restrictive transfusion strategy in younger and less severely ill ICU patients [4].

As detailed description of current transfusion practice in septic shock is lacking, our aim was to describe current clinical practice, including patient characteristics, potential triggers and outcome associated with RBC transfusion in unselected ICU patients with septic shock.

## Methods

### Design

We conducted a prospective cohort study of RBC transfusion of all adult septic shock patients in seven general ICUs in four university hospitals and three regional hospitals during a 5-month study period. The study was observational representing current practice.

### Ethics

The National Board of Health, the Ethics Committee of the Capital Region and the Danish Data Protection Agency approved the study, which was done according to the declaration of Helsinki. All measurements and interventions were clinically indicated, so consent was waived.

### Patient enrolment

Two of the authors (RGR and MUH) screened all patients admitted to five of the participating units in the study period; in two units local investigators screened the patients. Those included were patients fulfilling the consensus criteria for septic shock: 1. Documented or suspected infection. 2. Two of the following SIRS criteria: temperature < 36 or > 38 degrees Celsius, leukocyte count < 4 or > 12 x 10^9^/l, respiratory frequency > 20 breaths/min, mechanical ventilation or PaO_2_ < 4.3 kPa or heart rate > 90 beats/min. 3. Systolic blood pressure < 90 mmHg or vasopressor infusions after initial fluid resuscitation [5].

Both patients diagnosed with septic shock before or after admittance to the ICU were included. Patients were excluded for any of the following reasons: Age < 18 years, not undergoing active treatment, burn patients and trauma patients during the first 24 hours of ICU admittance. One member of Jehovah’s Witnesses was excluded from the study because of documented wish against transfusion.

### Data acquisition

Data were recorded for the day of admittance to the ICU, and for all days in septic shock by RGR and MUH. If RBCs were transfused at any shock day, data were registered for each transfusion episode. Data were registered on paper case report forms at the respective ICUs and merged into the study database by two investigators. The following baseline characteristics were recorded: Age, gender, type of admission, simplified acute physiology score (SAPS) II, source of infection, presence of acute or chronic cardiovascular disease (CVD) or chronic obstructive pulmonary disease (COPD). Cardiovascular disease was defined as previous ischemic heart disease, atherosclerosis (any location) or acute myocardial infarction (present if noted in patient files by the clinicians).

The daily registrations during shock included severity organ failure assessment (SOFA) score, minimum values of haemoglobin, arterial pH and ScvO_2_, highest concentration of plasma lactate and value of INR, lowest mean arterial pressure (MAP), highest heart rate (HR), maximum infusion rate of noradrenalin and volume of resuscitation fluid (isotonic saline, Ringers solutions and any colloid solutions) and volumes of blood products (RBCs, fresh frozen plasma and platelets).

For every transfusion episode, time, RBC volume and number of RBC units given were registered in addition to the following characteristics two hours prior to the transfusion: minimum values of haemoglobin, arterial pH, ScvO_2_ and PaO_2_/FiO_2_, maximum doses of vasopressor and inotropic agents, highest concentration of plasma lactate, lowest MAP and highest heart rate and if bleeding occurred as documented in patient notes. Bleeding during surgery and 24 hours postoperatively was not registered. If the time of RBC transfusion was not registered in the patient’s transfusion file or observation chart, the time of delivery from the blood bank was used instead. RBC transfusions given prior to ICU admittance, during surgery and within the first 24 hours postoperatively were not recorded.

Finally, the number of days in shock and in ICU and 28- and 90-day mortality were recorded, the latter by the use of the civil personal registration number in the administrative system for Danish hospitals (GS Open).

### Statistics

The primary analyses were to compare characteristics between RBC transfused and non-transfused patients. To describe any time-dependency or competing risk in relation to RBC transfusion, shock characteristics and co-interventions were compared for transfused and non-transfused patients on each shock day from day 1 to 5.

Continuous variables were expressed as medians with interquartile ranges (IQRs) and categorical variables as numbers with percentages of the total. Data were analysed using Wilcoxon’s rank sum test and chi-square test. Fisher’s exact test was used if the sample was five or below. Clinical characteristics of patients with and without CVD or bleeding were compared using a univariate mixed model allowing for correlations for multiple transfusions given to the same individual.

We examined the association between RBC transfusion and 90-day mortality with uni- and multivariate logistic regression. SAPS II, SOFA-score on day 1 and study site were used as covariates if they seemed to be associated with 90-day mortality in univariate analysis (p < 0.10). Medical vs. surgical patient category was forced into the model even though the p-value for the association with mortality was 0.12, because this characteristic appeared to differ between transfused and non-transfused patients (Table 1).

**Table 1:** Characteristics and outcomes of the 213 consecutive ICU patients with septic shock dependent on RBC transfusion during shock

**Characteristics**	**All patients**	**RBC transfused**	**Non-transfused**	**P value**
Number of patients	213	95 (45)	118 (55)	
Age	69 (60-77)	69 (60-76)	69 (59-77)	0.91
Female	87 (41)	38 (40)	49 (42)	0.82
Type of admission				0.03
Medical	124 (58)	64 (67)	60 (51)	
Emergency surgery	82 (39)	30 (32)	52 (44)	
Elective surgery	7 (3)	1 (1)	6 (5)	
Focus of infection				0.51
Pulmonary	81 (38)	39 (41)	42 (36)	
Gastrointestinal	68 (32)	25 (26)	43 (36)	
Soft tissue	20 (9)	8 (8)	12 (10)	
Urinary tract	9 (4)	6 (6)	3 (3)	
Other	15 (7)	7 (7)	8 (7)	
Unknown	20 (9)	10 (11)	10 (8)	
SAPS II	51 (40-65)	56 (45-69)	48 (37-61)	0.0005
Acute or chronic cardiovascular disease^1^	122 (57)	54 (57)	68 (58)	0.91
COPD	40 (19)	20 (21)	20 (17)	0.45
*Outcomes*				
Days in shock	3 (2-6)	5 (3-10)	2 (2-4)	< 0.0001
Days in ICU	6 (3-12)	10 (4-19)	4 (2-8)	< 0.0001
28-day mortality	102 (48)	56 (59)	46 (39)	0.004
90-day mortality	114 (54)	63 (66)	51 (43)	0.0008

Data are number of patient (%) or medians (IQRs).

COPD, chronic obstructive pulmonary disease; ICU, intensive care unit; IQR, interquartile range; SAPS II, simplified acute physiology score II.

^1^Only 9 patients had acute cardiovascular disease, 5 were transfused and 4 were not.

All analyses were performed using SAS version 9.2 (SAS Institute Inc., Cary, NC) and values of p< 0.05 were considered statistically significant. P-values between 0.01 and 0.05 were interpreted with caution due to multiple comparisons.

### Handling of missing data

In general, there were very few missing data and all analyses presented were complete case analyses. All patients were included in the multivariate analysis and we used the obtained SAPS II and SOFA score. Thus, we did not impute missing score components.

## Results

Two-hundred-thirteen patients with septic shock were included of who 139 were from university hospitals and 74 from regional hospitals. Their characteristics are given in Table 1. Twenty-nine (14%) patients had at least one bleeding episode registered and these occurred mainly on days 1 and 2 (Table 2).

**Table 2:** Clinical characteristics dependent on RBC transfusion on consecutive days of septic shock

**Day 1**	**RBC**	**No RBC**	**P value**
Number of patients (%)	34 (16)	179 (84)	-
Bleeding episode, n (%)	7 (21)	0	< 0.0001
SOFA	12 (9-14)	9 (7-12)	0.0004
NA dose (max) (μg/kg/min)	0.20 (0.07-0.35)	0.15 (0.06-0.30)	0.27
Haemoglobin (min) (g/dl)	7.3 (6.8-7.9)	10.0 (8.9-11.1)	< 0.0001
ScvO_2_ (min) (%)	68 (51-76), n = 16	71 (65-76), n = 83	0.21
Lactate (max) (mmol/l)	3.1 (1.5-7.9)	2.8 (1.7-4.6), n = 178	0.39
Day 2			
Number of patients (%)	32 (17)	156 (83)	-
Bleeding episode, n (%)	8 (25)	0	< 0.0001
SOFA	9 (8-13)	9 (7-13)	0.91
NA dose (max) (μg/kg/min)	0.13 (0.08-0.22)	0.15 (0.07-030)	0.49
Haemoglobin (min) (g/dl)	7.9 (7.6-8.7)	9.7 (8.9-10.5)	< 0.0001
ScvO_2_ (min), (%)	71 (68-75), n = 21	72 (65-76), n = 68	0.85
Lactate (max) (mmol/l)	2.0 (1.3-4.1)	2.3 (1.6-3.3), n = 155	0.72
Day 3			
Number of patients (%)	31 (24)	98 (76)	-
Bleeding episode, n (%)	4 (13)	0	0.0003
SOFA	10 (9-14)	10 (7-12)	0.21
NA dose (max) (μg/kg/min)	0.16 (0.09-0.25)	0.13 (0.08-0.26)	0.39
Haemoglobin (min) (g/dl)	8.2 (7.4-8.5)	9.7 (9.0-10.3)	< 0.0001
ScvO_2_ (min) (%)	67 (63-72), n = 18	72 (64-75), n = 38	0.15
Lactate (max) (mmol/l)	2.0 (1.5-3.0)	1.9 (1.4-2.6), n = 97	0.45
Day 4			
Number of patients (%)	17 (17)	82 (83)	-
Bleeding episode, n (%)	3 (18)	0	0.0001
SOFA	12 (9-14)	10 (8-14)	0.34
NA dose (max) (μg/kg/min)	0.13 (6-25)	0.10 (6-19)	0.43
Haemoglobin (min) (g/dl)	8.4 (7.9-8.7)	9.3 (8.5-10.0)	0.0004
ScvO_2_ (min) (%)	70 (65-72), n = 10	66 (63-76), n = 20	0.80
Lactate (max) (mmol/l)	1.7 (1.2-2.4)	1.7 (1.3-2.7), n = 81	0.60
Day 5			
Number of patients (%)	18 (24)	58 (76)	-
Bleeding episode, n (%)	4 (22)	0	0.0002
SOFA	14 (12-16)	10 (8-13)	0.01
NA dose (max) (μg/kg/min)	0.14 (0.08-0.40)	0.12 (0.04-0.20)	0.08
Haemoglobin (min) (g/dl)	7.7 (7.1-8.5)	9.5 (8.7-10.3)	< 0.0001
ScvO_2_ (min) (%)	74 (69-78), n = 6	67 (63-77), n = 19	0.30
Lactate (max) (mmol/l)	2.3 (1.5-3.3)	1.7 (1.4-2.3)	0.33

Values are as medians (interquartile ranges) of those patients with registered data; n is given in the cells, if not all patients had the value registered.

Min, minimum; max, maximum; n, number; NA, noradrenalin; SOFA, sequential organ failure assessment; ScvO_2_, central venous oxygen saturation.

### RBC transfusion

Ninety-five (45%) of the patients received a total of 398 RBC units during 315 registered RBC transfusion episodes with a median of 3 (2-5) transfused units per patient. Seventy-seven % of the transfusions were administrated as single units, 20% as two units and 3% as three or more units.

The RBC transfused group had higher SAPS II, but there were no differences in age and the frequencies of CVD or COPD between the groups (Table 1).

In the first 5 days of shock the transfusion frequency varied between 16 - 24%. The occurrence of bleeding and daily minimal haemoglobin concentration were the only factors that consistently differed between RBC transfused and non-transfused patients (Table 2). SOFA scores were higher in RBC transfused compared to non-transfused patients on days 1 and 5 (Table 2). In contrast, there were no differences on any days in minimum ScvO_2_ or maximum dose of noradrenalin or concentration of lactate between the groups. These data were largely unchanged by the exclusion of bleeding patients (Additional file 1: Table S1).

More crystalloids and colloids were given on days 1 and 2, but there were no differences between RBC transfused and non-transfused patients (Table 3). The RBC transfused group received significantly more platelets on days 1, 3 and 5 and significantly more fresh frozen plasma on day 1 (Table 3).

**Table 3:** Daily use of crystalloids, colloids and blood products depending on RBC transfusion on consecutive days of septic shock

**Day 1**	**RBC**	**No RBC**	**P value**
Number of patients (%)	34 (16)	179 (84)	-
RBCs (ml)	490 (300-735)	-	-
Crystalloids and colloids (ml)	3000 (1800-4600), n = 31	3000 (1600-5000), n = 165	0.62
Platelets (ml)	350 (300-370), n = 7	350 (350-1050), n = 11	0.009
Fresh frozen plasma (ml)	550 (540-1080), n = 13	545 (531-600), n = 24	0.0004
Day 2			
Number of patients (%)	32 (17)	156 (83)	-
RBCs (ml)	490 (245-490)	-	-
Crystalloids and colloids (ml)	2000 (1250-3000), n = 29	1650 (1000-3000), n = 129	0.22
Platelets (ml)	550 (420-980), n = 4	700 (350-1050), n = 13	0.49
Fresh frozen plasma (ml)	1050 (540-1200), n = 5	600 (540-1067), n = 16	0.37
Day 3			
Number of patients (%)	31 (24)	98 (76)	-
RBCs (ml)	245 (245-490)	-	-
Crystalloids and colloids (ml)	1325 (800-2400), n = 22	1400 (900-2000), n = 71	0.91
Platelets (ml)	350 (350-1050), n = 8	350 (350-1050), n = 5	0.001
Fresh frozen plasma (ml)	540 (408-680), n = 8	540 (540-813), n = 11	0.06
Day 4			
Number of patients (%)	17 (17)	82 (83)	-
RBCs (ml)	245 (245-245)	-	-
Crystalloids and colloids (ml)	1500 (1000-2000), n = 9	1000 (500-1300), n = 41	0.40
Platelets (ml)	700 (525-1050), n = 4	350 (300-700), n = 10	0.18
Fresh frozen plasma (ml)	405 (270-540), n = 2	560 (540-600), n = 10	0.88
Day 5			
Number of patients (%)	18 (24)	58 (76)	-
RBCs (ml)	245 (245-490)	-	-
Crystalloids and colloids (ml)	500 (200-1100), n = 14	1000 (900-2000), n = 37	0.45
Platelets (ml)	350 (350-700), n = 5	1050, n = 1	0.0009*
Fresh frozen plasma (ml)	n = 0	537 (524-550), n = 2	0.44

Values are as medians (interquartile ranges) of those patients with registered data; n is given in the cells, if not all patients had the value registered.

*Significantly more platelets were given in the RBC group as 5 patients received platelets vs. only 1 in the No RBC group.

RBCs, red blood cells.

### RBC triggers

**Figure 1:**
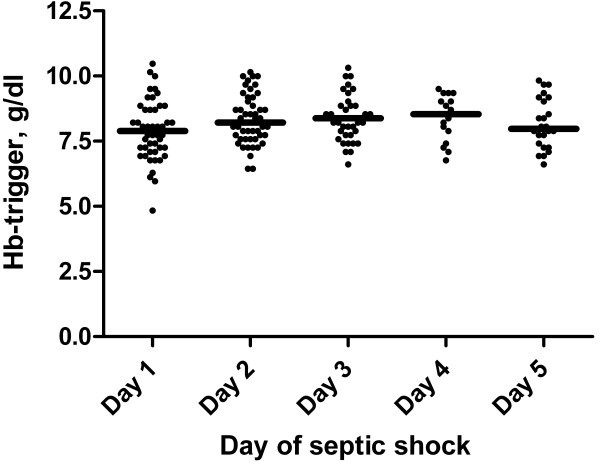
**Haemoglobin (Hb) trigger levels in septic shock.** The scatter plot depicts the Hb levels within two hours before RBC transfusion on days of shock. The bars denote medians.

The median value of the pre-transfusion haemoglobin was 8.1 g/dl (7.4-8.9) and did not differ over time (Figure 1). Patients with CVD were transfused at a higher haemoglobin level than those without CVD (Table 4). At time of transfusion, patients with bleeding had higher concentration of lactate and lower oxygenation ratio compared to those without bleeding (Table 4).

**Table 4:** Differences in potential physiological triggers in RBC-transfused patients 0-2 hours prior to transfusion depending on cardiovascular disease or bleeding

	**Differences, bleeding vs. non-bleeding**	**Standard error**	**P value**
Haemoglobin (g/dl)	0.2	0.1	0.19
pH	-0.03	0.01	0.04
PaO_2_/FiO_2_ (kPa)	-5.4	1.5	0.0003
ScvO_2_ (%)	-5.2	3.3	0.12
Lactate (max) (mmol/l)	1.5	0.4	0.0005
MAP (min) (mmHg)	-2	2	0.19
Heart rate (max) (beats/min)	-3	3	0.38
	**Differences, CVD vs. no CVD**	**Standard error**	**P value**
Haemoglobin (g/dl)	0.4	0.2	0.048
pH	0	0.02	0.90
PaO_2_/FiO_2_	-3.6	2.1	0.10
ScvO_2_ (%)	-2.7	3.3	0.43
Lactate (max) (mmol/l)	-0.7	0.4	0.23
MAP (min) (mmHg)	0	2	0.98
Heart rate (max) (beats/min)	-4	5	0.40

CVD, cardiovascular disease; MAP, max, maximum; mean arterial pressure; min, minimum; ScvO_2_, central venous oxygen saturation.

Of the 315 registered RBC transfusion episodes only 39 (12%) had registered measurements of ScvO_2_ in the 2-hour period preceding transfusion. No patients had a catheter for continuous ScvO_2_ measurement.

### Outcome

RBC transfused patients had more days in shock and longer ICU stay compared to the non-transfused (Table 1). At discharge from the study ICU 9% of patients were discharged to another ICU, 61% to a hospital ward and 30% of the patients died in the ICU. The 90-day mortality rate was 54% in the full cohort; 66% in the RBC transfused vs. 43% in the non-transfused patients (p = 0.0008) (Table 1).

Univariate logistic regression analyses (Additional file 1: Table S2) showed that RBC transfusion, SAPS II and SOFA-score at day 1 significantly associated with 90-day mortality whereas study site did not (RBC transfusion vs. no RBC transfusion: odds ratio (OR) for death 2.59 (95% CI 1.48 - 4.53), p = 0.0009). In the adjusted multivariate analysis (Additional file 1: Table S2), RBC transfusion was not associated with 90-day mortality (RBC transfusion vs. no RBC transfusion: OR for death 1.72 (95% CI 0.91 - 3.24), p = 0.10).

## Discussion

The main findings in this study were that 45% of the septic shock patients were transfused during shock and that these patients had more bleeding episodes and higher disease severity on admission (SAPS II and SOFA score). Bleeding and haemoglobin concentration were the only factors that consistently differed between transfused and non-transfused throughout the course of shock, but the majority of transfusions were given to non-bleeding patients. Patients with bleeding had higher lactate levels and worse oxygenation at time of transfusion, compared to those without bleeding, but the haemoglobin levels did not differ between the groups. The median pre-transfusion haemoglobin value was 8.1 g/dl in all transfused patients, and patients with CVD were transfused at a higher haemoglobin level than those without CVD. Overall RBC transfused patients also received more plasma and platelets and had more days in shock, longer ICU stay and markedly higher 90-day mortality than those not transfused. The association between RBC transfusion and mortality did not persist after adjustment in the multivariate logistic regression analysis.

The transfusion of RBCs in non-bleeding patients should be based on the need for increased oxygen delivery and the haemoglobin level [6]. Our findings indicate that the haemoglobin level was the prevailing trigger factor for RBC transfusion. This observation is comparable to those of a post hoc analysis of shock patients in a cohort of acute lung injury patients and those of a less detailed study in septic shock [7,8]. Our transfusion rate of 45% was in the higher end compared to those observed in other critical care patients (25% to 53%) [9] and in community-acquired severe sepsis (39%) [10]. The pre-transfusion value of haemoglobin of 8.1 g/dl in our study was lower than in the previous cohorts of general ICU patients (8.4 to 8.6 g/dl [4,11-13]), but slightly higher than in community-acquired severe sepsis (7.7 g/dl) [10]. Referring to the SSC guidelines, one would expect to find lower ScvO_2_ and a higher haemoglobin trigger values on day 1 compared to the following days, but this was not the case. So RBC transfusion practice did not appear to adhere to the SSC guidelines neither in this nor in the previously published cohorts of patients with septic shock [7,8].

The SSC guidelines are based on the results of two RCTs with potentially divergent results [3,4], but neither of these trials may fully inform ICU clinicians on when to transfuse RBCs in the septic shock patients. TRICC included general ICU patients after initial stabilization. In the sub-group analysis of patients with severe sepsis [4], liberal RBC transfusion may have increased the relative risk of death by 30%, but this did not reach statistical significance. The EGDT trial showed remarkable effects of a complex protocol in the early phase of resuscitation of patients with severe sepsis including RBC transfusion in the case of persistent hypoperfusion in a single US emergency department [3]. Therefore it is difficult to assess the sole effect of RBC transfusion in ICU patients with septic shock. Furthermore, neither of these trials used pre-storage leukocyte-depleted RBCs, which is now the standard in most centres. This may also have affected the outcome [11].

RBC transfusion has been identified as a risk factor for mortality in multiple epidemiological studies of general ICU patients [8,12-16] and in a systematic review of cohort studies of RBC transfusion in critical care patients in general [17]. However, neither Parson et al. [8] nor the SOAP study collaborators found that RBC transfusion was significantly associated with increased mortality using multivariate analysis [14]. The same was the case in our data, where the point estimate indicated harm by RBC transfusion, but the confidence interval crossed 1.0. Moreover when patients were matched by propensity scoring in the SOAP cohort, RBC transfusion was associated with improved outcome [14]. These results are supported by data from a single, surgical ICU [15] and a cohort of patients with community-acquired severe sepsis from multiple ICUs [10]. It may be questioned if any of the statistical models used are valid to adjust for the obvious differences between transfused and non-transfused in cohorts of ICU patients. There are problems at multiple levels. First, the most valid adjustments are done with baseline variables. In ICU cohorts first day SAPS II, APACHE II and SOFA scores are often used, but these are not true baseline variables, because they are calculated from data from the first 24 hours of intensive care. The problem is that RBC transfusion given in these 24 hours in ICU may influence the scores, which is very obvious for APACHE II scoring where the lowest haematocrit value is included. Other variables in these scores may also be affected by any positive or negative effects of RBCs which may invalid the adjustment. Second, neither of the scoring systems have 100% predictive power for mortality, so that confounding by indication may remain in spite of adjustment for disease severity using these scores. Third, transfusion is often a repeated intervention and the reason for transfusion may change over time and the time ‘at risk’ also varies. Our study indicate that bleeding and haemoglobin level was the main trigger in this cohort, but also SOFA score on day 1 and 5 differed between RBC transfused and non-transfused, whereas we observed no differences on day 2, 3 and 4. Other unmeasured characteristics may also have differed between the groups making it difficult to correct differing patient characteristics at repeated transfusions over time. Lastly, competing risk may exist as shown in our cohort, where RBC transfused patients were more likely to receive other blood products. Controlling for all these potential confounders may be very difficult even with complex statistical modelling. We believe that the only adequate method to assess the safety and efficacy of RBC transfusion in patients with septic shock is performing large RCTs testing different triggers and levels preferable in multiple trials. The potential for such RCTs was shown in patients with acute upper-gastrointestinal bleeding where RBC transfusion at haemoglobin levels of 7 vs. 9 g/dl improved survival [18]. In patients with septic shock, the on-going TRISS – Transfusion-Requirements in Septic Shock – trial (NCT01485315) is to randomise 1,000 patients in 30 Scandinavian ICUs through the Scandinavian Critical Care Trials Group [19]. The TRISS trial will test if using haemoglobin levels of 7 vs. 9 g/dl as RBC transfusion trigger will alter 90-day mortality and the degree of organ failure. The interim analysis was done in June 2013 after 90-day follow-up of 500 patients and the trial is expected to be finalized early in 2014.

### Strengths of the study

The strengths of our study include prospective inclusion of all consecutive patients at multiple university and non-university ICUs. A specific patient group with expected high risk/benefit was included using well-established definitions, and follow-up was done through the national patient registry using civil registration numbers, which ensured full follow-up. Using detail registration and reporting of both baseline and time-dependent characteristics allowed us to detail transfusion practice. The mortality rate was high as observed in recent pragmatic trials including patients with septic shock [20,21].

### Limitations of the study

The sample size was relatively small and obtained in one country only. This was an observational study and parameters were registered from observational charts, thus the data registration was based upon the assumption that all observations were registered. The lack of full electronic source data may have hampered data acquisition. Furthermore, the routine of registering the exact time for administrating RBC varied among ICUs, and we made no distinction between administration and prescription of RBCs, which does not necessarily correspond in time. Finally, blood age and the periods before and after ICU admittance are all likely to be of importance, but these data were not included in our study due to logistical and financial reasons.

## Conclusions

Half of the patients received RBCs during septic shock and these patients were more severely ill at admission and had more bleeding episodes and more organ failures. Haemoglobin level was the only measure that consistently differed between transfused and non-transfused patients.

## Abbreviations

APACHE: Acute physiology and chronic health evaluation; CI: Confidence interval; COPD: Chronic obstructive pulmonary disease; CVD: Cardiovascular disease; EGDT: Early goal directed therapy; FiO2: Fractional inspired oxygen; HR: Heart rate; ICU: Intensive care unit; IQR: Interquartile range; MAP: Mean arterial pressure; OR: Odds ratio; PaO2: Partial pressure of oxygen in arterial blood; RBC: Red blood cell; RCT: Randomized clinical trial; SAPS: Simplified acute physiology score; ScvO2: Central venous oxygen saturation; SIRS: Systemic inflammatory response syndrome; SOFA: Sequential organ failure assessment; SSC: Surviving sepsis campaign; TRICC: Transfusion requirements in critical care

## Competing interests

The study was supported by the Danish Strategic Research Council. The Department of Intensive Care, Rigshospitalet receives support for research from CSL Behring, Fresenius Kabi, BioPorto and Cosmed. Anders Perner was sponsor-investigator of the 6S trial, which was supported by B Braun Medical, and has received honoraria from Ferring Pharmaceutical (trial steering committee member) and LPB S.A. (speaker fee). None of the other authors have conflicts of interests.

## Authors’ contributions

MH and RR contributed equally to study design, data acquisition, statistical analyses, and interpretation and also made the first draft of the manuscript. NH participated in the statistical analyses, data interpretation and manuscript revision. AP participated in the study design, data interpretation and manuscript revision. The remaining authors participated in data acquisition at the respective ICUs. All authors read and approved the final manuscript.

## Supplementary Material

Additional file 1: Table S1 Clinical characteristics dependent on RBC transfusion on consecutive days of septic shock in patients without bleeding. **Table S2.** Results of the logistic regression model of risk factors for death in ICU for patients with septic shock. Click here for file
