# Supplementary material for: Red blood cell transfusion in septic shock - clinical characteristics and outcome of unselected patients in a prospective, multicentre cohort
Source: Scand J Trauma Resusc Emerg Med. 2014 Feb 27;22:14. doi: 10.1186/1757-7241-22-14 (PMC3938972; doi:10.1186/1757-7241-22-14)
Supplement: Additional file 1: Table S1 — Clinical characteristics dependent on RBC transfusion on consecutive days of septic shock in patients without bleeding. Table S2. Results of the logistic regression model of risk factors for death in ICU for patients with septic shock. [file 1757-7241-22-14-S1.docx]

| **Supplementary table 1** Clinical characteristics dependent on RBC transfusion on consecutive days of septic shock in patients without bleeding | | | | | |
| --- | --- | --- | --- | --- | --- |
| Day 1 |  | RBC | No RBC | P value |  |
| Number of patients (%) |  | 27 (13) | 179 (87) | - |  |
| SOFA |  | 11 (9-16) | 9 (7-12) | 0.001 |  |
| NA dose (max) (µg/kg/min) |  | 0.18 (0.06-0.35) | 0.15 (0.06-0.30) | 0.54 |  |
| Haemoglobin (min) (g/dl) |  | 7.4 (7.1-7.9) | 10.0 (8.9-11.1) | < 0.0001 |  |
| ScvO_2_ (min) (%) |  | 69 (53-76), n=12 | 71 (65-76), n=83 | 0.36 |  |
| Lactate (max) (mmol/l) |  | 2.8 (1.2-5.2) | 2.8 (1.7-4.6), n=178 | 0.88 |  |
| Day 2 |  |  |  |  |  |
| Number of patients (%) |  | 24 (13) | 156 (87) | - |  |
| SOFA |  | 9 (8-11) | 9 (7-13) | 0.78 |  |
| NA dose (max) (µg/kg/min) |  | 0.11 (0.08-0.22) | 0.15 (0.07-030) | 0.45 |  |
| Haemoglobin (min) (g/dl) |  | 7.9 (7.4-8.7) | 9.7 (8.9-10.5) | < 0.0001 |  |
| ScvO_2_ (min), (%) |  | 71 (64-76), n=16 | 72 (65-76), n=68 | 0.69 |  |
| Lactate (max) (mmol/l) |  | 1.6 (1.3-2.5) | 2.3 (1.6-3.3), n=155 | 0.04 |  |
| Day 3 |  |  |  |  |  |
| Number of patients (%) |  | 27 (22) | 98 (78) | - |  |
| SOFA |  | 10 (8-14) | 10 (7-12) | 0.58 |  |
| NA dose (max) (µg/kg/min) |  | 0.16 (0.09-0.23) | 0.13 (0.08-0.26) | 0.52 |  |
| Haemoglobin (min) (g/dl) |  | 8.2 (7.6-8.5) | 9.7 (9.0-10.3) | < 0.0001 |  |
| ScvO_2_ (min) (%) |  | 68 (61-72), n=16 | 72 (64-75), n=38 | 0.20 |  |
| Lactate (max) (mmol/l) |  | 2.0 (1.5-2.7) | 1.9 (1.4-2.6), n=97 | 0.63 |  |
| Day 4 |  |  |  |  |  |
| Number of patients (%) |  | 14 (15) | 82 (85) | - |  |
| SOFA |  | 12 (9-13) | 10 (8-14) | 0.54 |  |
| NA dose (max) (µg/kg/min) |  | 0.12 (5-20) | 0.10 (6-19) | 0.74 |  |
| Haemoglobin (min) (g/dl) |  | 8.5 (7.4-8.7) | 9.3 (8.5-10.0) | 0.001 |  |
| ScvO_2_ (min) (%) |  | 71 (67-73), n=8 | 66 (63-76), n=20 | 0.78 |  |
| Lactate (max) (mmol/l) |  | 1.6 (1.2-1.9) | 1.7 (1.3-2.7), n=81 | 0.38 |  |
| Day 5 |  |  |  |  |  |
| Number of patients (%) |  | 14 (19) | 58 (81) | - |  |
| SOFA |  | 14 (12-16) | 10 (8-13) | 0.04 |  |
| NA dose (max) (µg/kg/min) |  | 0.13 (0.08-0.44) | 0.12 (0.04-0.20) | 0.12 |  |
| Haemoglobin (min) (g/dl) |  | 8.1 (7.3-8.5) | 9.5 (8.7-10.3) | < 0.0001 |  |
| ScvO_2_ (min) (%) |  | 70 (68-75), n=4 | 67 (63-77), n=19 | 0.66 |  |
| Lactate (max) (mmol/l) |  | 1.6 (1.5-3.2) | 1.7 (1.4-2.3) | 0.78 |  |
| Values are as medians (interquartile ranges) of those patients with registered data; n is given in the cells, if not all patients had the value registered.  Min, minimum; max, maximum; n, number; NA, noradrenalin; SOFA, sequential organ failure assessment; ScvO_2_, central venous oxygen saturation. | | | | | |

**Supplementary table 2**

**Results of the logistic regression model of risk factors for death in ICU for patients with septic shock**

| **Variable** | **Univariate analysis** | | **Multivariate analysis** | |
| --- | --- | --- | --- | --- |
|  | Odds ratio  (95%-CI) | P-value | Odds ratio  (95%-CI) | P-value |
| Blood transfusion vs. no blood transfusion | 2.59 (1.48-4.53) | <0.001 | 1.72 (0.91-3.24) | 0.10 |
| Medical vs. surgical reason for admission | 1.55 (0.90-2.68) | 0.12 | 1.17 (0.62-2.18) | 0.63 |
| Study site | - | 0.24 | - | - |
| Transformed SAPS II | <0.001  (<0.001-<0.001) | <0.0001 | <0.001  (<0.001-<0.001) | <0.0001 |
| Transformed SOFA at day 1 | <0.001  (<0.001-<0.001) | <0.0001 | 0.008  (<0.001-150.1) | 0.33 |

Inverse transformations were used for SAPS and SOFA scores to obtain an acceptable linearity. The odds ratio estimates correspond to a one unit increase in the inverse score, which is less clinical meaningful.

Study site was not included in the multivariate model due to the relatively high P-value in the univariate model.

There was no significant interaction between receiving blood and the other covariates in the multivariate model.
